# Bladder carcinosarcoma with rhabdomyoblastic differentiation: a rare case report

**DOI:** 10.1093/jscr/rjac206

**Published:** 2022-05-05

**Authors:** Moez Rahoui, Kheireddine Mrad Dali, Kays Chaker, Yassine Ouanes, Mokhtar Bibi, Ahmed Sellami, Sami Ben Rhouma, Yassine Nouira

**Affiliations:** Urology Department, La Rabta Hospital, Tunis, Tunisia; Urology Department, La Rabta Hospital, Tunis, Tunisia; Urology Department, La Rabta Hospital, Tunis, Tunisia; Urology Department, La Rabta Hospital, Tunis, Tunisia; Urology Department, La Rabta Hospital, Tunis, Tunisia; Urology Department, La Rabta Hospital, Tunis, Tunisia; Urology Department, La Rabta Hospital, Tunis, Tunisia; Urology Department, La Rabta Hospital, Tunis, Tunisia

## Abstract

Carcinosarcoma is a distinct neoplasm consisting of bidirectional differentiation toward epithelial and mesenchymal cells. Bladder localization is rare and the association with a rahbdomyoblastic component is exceptional. Few cases of bladder carcinosarcoma with rhabdomyoblastic differentiation have been reported in the literature. We present a case of a bladder carcinosarcoma in a 68-year-old man who presented with terminal hematuria and discuss difficulties of diagnostic and treatment.

## INTRODUCTION

Carcinosarcoma is a rare tumor [1]. The diagnosis is usually made at an advanced stage and the prognosis is poor. The bladder localization is rare and the tumor represents 0.3% of bladder tumors [1]. The association with rhabdomyoblastic differentiation is exceptional. No reference treatment can be proposed, although some long-time survival has been observed with total cystectomy. In this article, we report a case of bladder carcinosarcoma showing distinct rhabdomyoblastic differentiation and we aimed to remind the clinical, histological and therapeutic features of this rare tumor.

## CASE REPORT

**Figure 1:**
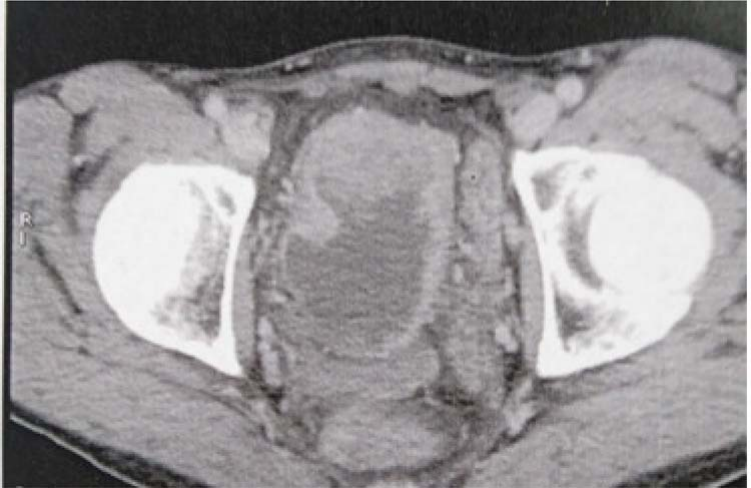
CT revealing a bladder tumor of 5 × 5 cm over the dome and anterior wall.

**Figure 2:**
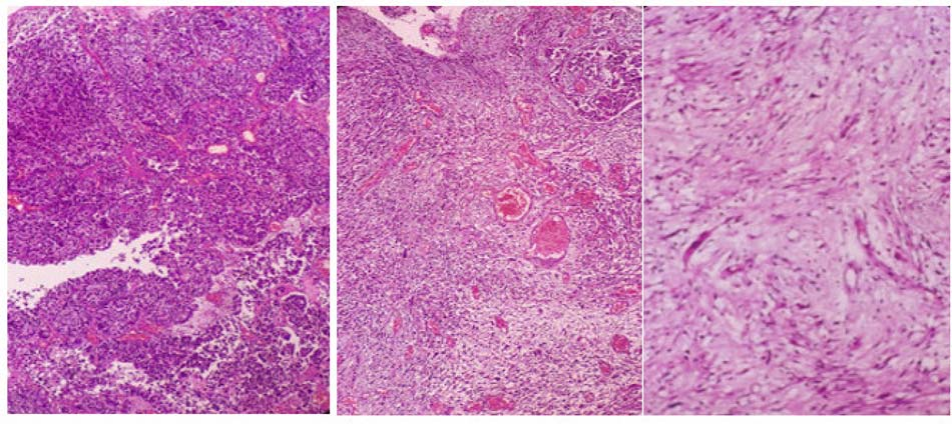
Microscopic overview of the malignant urothelial tumor with sarcomatoid and rhabdomyoblastic components (hematoxylin and eosin ^*^ 20).

**Figure 3:**
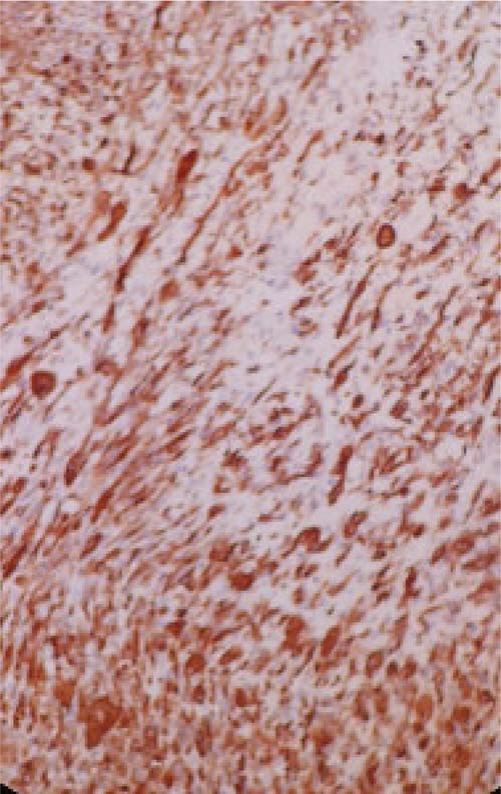
Immunohistochemical (IHC) study shows the positivity of rhabdomyoblastic cells to the anti-desmin antibody (IHC ^*^ 20).

A 68-year-old man, diabetic, presented for terminal hematuria associated with pelvic pain. His physical examination was normal. Ultrasonography and chest and abdominopelvic computed tomography (CT) scan revealed a urinary bladder tumor of 5 × 5 cm over the dome and the anterior wall with normal upper tract (Fig. 1). Cystoscopic exploration revealed a solid lesion in the anterior wall and the dome with a large base. The patient underwent a complete and deep transurethral resection of the bladder tumor. Anatomopathological examination showed a carcinosarcoma appearance with a rhabdomyoblastic differentiation (Figs 2 and 3). A chest and abdominopelvic CT scan, with intravenous administration of contrast medium (CT), did not show pelvic lymphadenopathy or secondary location. Radical surgery was then decided without neoadjuvant therapy. Cyst prostatectomy with Bricker diversion and lymph node dissection was performed. The postoperative course was uneventful. The pathological examination of the surgical specimen confirms the diagnosis of a bladder carcinosarcoma with a rhabdomyoblastic differentiation. After 12 months of clinical and radiological check-ups, there was no functional complaint or any sign of reoccurrence.

## DISCUSSION

Carcinosarcoma of the bladder is a high-stage tumor at diagnosis and has a poor prognosis**.** The term ‘carcinosarcoma’ is used to refer to a malignant neoplasm whose overt epithelial histology is mixed with distinctive mesenchymal histology with heterologous elements [1]. The preferential location of this tumor is the uterus in women and the bladder in men [2]. The histological features of carcinosarcomas of the bladder are variable, macroscopically, they can be nodular, large or polypoid. The most common sarcomatous elements are chondrosarcoma, leiomyosarcoma and malignant fibrous histiocytoma [2]. In our case, we noted a rhabdomyoblastic differentiation. The etiology of this tumor is not yet clearly defined but the history of previous radiotherapy or chemotherapy could be responsible [2]. These tumors are more common in men than women, with a ratio of 4:1 [2]. Bladder carcinosarcoma is an aggressive tumor and there is no consensus about its treatment. Several treatments have been proposed but it turns out that a multimodal treatment seems necessary. Some authors have proposed transurethral resection associated with a partial cystectomy [3]. However, others have shown that radical cystectomy represents the reference treatment [4]. Neoadjuvant/adjuvant radio chemotherapy has been used in many cases, and there were complete responses after neoadjuvant treatment [4]. However, there is no reference treatment. Surgery represents the best therapeutic option with better overall survival. In a retrospective study that analyzed 221 cases, the overall 5-year cancer-specific survival rate after radical cystectomy was 20.3% [1,5]. In the present case, the patient has survived 12 months without recurrence.

## CONCLUSION

Bladder carcinosarcoma is an aggressive tumor and has a poor prognosis. Radical cystectomy represents the best therapeutic option with better overall survival. Sometimes, external radiotherapy can be offered.

## CONFLICT OF INTEREST STATEMENT

The authors declare that there are no conflicts of interest regarding the publication of this article.

## FUNDING

We have no financial sources for our research.
